# Iatrogenic Bile Duct Injury in 2025: Real World Causes, Referral Patterns, and Reconstruction Outcomes From a Tertiary Care Centre in a Sub-himalayan Region

**DOI:** 10.7759/cureus.106637

**Published:** 2026-04-08

**Authors:** Vipan Kumar, Anupam Jhobta, Puneet Mahajan, Rajesh Sharma

**Affiliations:** 1 Surgery, Army Hospital Research & Referral, Delhi, IND; 2 Surgery / Surgical Gastroenterology, Indira Gandhi Medical College & Hospital, Shimla, IND; 3 Radiodiagnosis, Indira Gandhi Medical College & Hospital, Shimla, IND; 4 General Surgery, Indira Gandhi Medical College & Hospital, Shimla, IND; 5 Gastroenterology, Indira Gandhi Medical College & Hospital, Shimla, IND

**Keywords:** biliovascular injury, conventional laparoscopic cholecystectomy, hepaticojejunostomy, laparoscopic cholecystectomy, post-cholecystectomy bile duct injury

## Abstract

Background

Despite widespread adoption of laparoscopic cholecystectomy (LC), major bile duct injuries (BDIs) continue to occur, particularly in real-world, resource-limited settings.

Methods

This retrospective study analysed a prospectively maintained BDI-benign biliary stricture registry at the hepatopancreatobiliary (HPB) and gastrointestinal (GI) surgery unit of Indira Gandhi Medical College (IGMC), Shimla, a tertiary HPB referral centre in the sub-Himalayan region. All adult patients referred between January 2020 and December 2024 with major iatrogenic BDI requiring definitive surgical reconstruction were included. Minor biliary injuries managed conservatively or endoscopically were excluded. Demographic data, injury characteristics, surgeon experience, referral patterns, management, and outcomes were evaluated.

Results

Twenty-three patients underwent Roux-en-Y hepaticojejunostomy for major BDIs. Most injuries occurred during LC (91.3%) and were associated with difficult gallbladder anatomy (73.9%). Seventeen injuries (73.9%) were caused by surgeons with less than three years of post-training experience (p = 0.028). Primary repair was attempted at the index centre in 43.5% of patients, frequently in high-grade injuries. Referral beyond 72 hours occurred in 91.3% of cases. Three additional patients with catastrophic vasculobiliary injury died before reconstruction. All reconstructed patients had favourable medium-term outcomes.

Conclusion

Major BDI remains largely preventable. Early referral, avoidance of unsafe repair, adoption of bailout strategies, structured early-career supervision, and improved infrastructure are essential to reduce preventable morbidity and mortality.

## Introduction

Bile duct injury (BDI) remains one of the most serious and life‑altering complications of cholecystectomy, with profound long‑term consequences for patients and significant medico‑legal, psychological, and financial implications for surgeons and healthcare systems [1-3].

Laparoscopic cholecystectomy (LC) is the gold‑standard operation for symptomatic gallstone disease and is among the most frequently performed abdominal procedures worldwide [4,5].

Despite more than three decades of global experience with LC, major BDIs continue to occur at a consistent and clinically relevant rate. Given the sheer volume of LCs performed annually, even a low incidence of BDI translates into thousands of affected patients each year, many of whom experience lifelong morbidity, repeated interventions, and impaired quality of life [6,7].

Injury severity may be further compounded by associated vascular damage, particularly involving the right hepatic artery or portal vein, converting a biliary injury into a catastrophic biliovascular event. It is essential to distinguish minor biliary complications from major BDI [8,9].

Minor injuries, such as cystic duct stump leaks or small peripheral duct leaks, are increasingly managed conservatively or endoscopically. In contrast, major BDI characterized by ductal transection, tissue loss, ischemia, or biliary discontinuity require complex surgical reconstruction, most commonly Roux‑en‑Y hepaticojejunostomy (HJ), and account for the greatest long‑term morbidity [7,10].

Although extensive literature exists on the management of BDI, most published data originate from high‑volume referral centres and do not adequately capture the real‑world circumstances under which these injuries occur.

In particular, the early independent practice phase immediately after completion of surgical training represents a vulnerable and under‑reported period. Surgeons during this phase often operate in peripheral or resource‑constrained environments with limited supervision and infrastructure [6,11].

Furthermore, a substantial proportion of BDIs never enter public datasets, as patients are selectively referred to apex centres, private centres, or managed locally, resulting in systematic underestimation of the true burden [12].

BDI should be regarded as an unintended surgical accident rather than a moral or professional failure. However, patient outcome is critically determined by the surgeon’s response once injury is suspected or recognized. Inappropriate decision‑making, including conversion to open surgery with attempted repair in non-hepatopancreatobiliary (HPB) settings, frequently escalates injury severity, compromises definitive reconstruction, and may result in fatal biliovascular damage [13,14].

Against this background, the present study analyzes five years of prospectively maintained data on major iatrogenic BDI requiring definitive surgical reconstruction at a tertiary HPB unit in the Sub‑Himalayan region. Minor biliary injuries managed conservatively or endoscopically were excluded.

The objectives were to identify causative factors, injury patterns, referral characteristics, and outcomes of reconstruction, while highlighting system‑level and training‑related gaps that continue to make major BDIs preventable in 2025.

## Materials and methods

This retrospective analysis was performed on a prospectively maintained BDI-benign biliary stricture registry at the HPB and gastrointestinal (GI) surgery unit of Indira Gandhi Medical College (IGMC), Shimla, a government tertiary care teaching hospital and the principal HPB referral centre for the Sub-Himalayan region.

All consecutive adult patients referred to our center between January 2020 and December 2024 with major iatrogenic BDI requiring surgical reconstruction were included. Data analysis was conducted in 2025. The centre receives referrals from government hospitals, private centres, and teaching institutions across Himachal Pradesh and adjoining states.

Adult patients aged 18 years or older with major iatrogenic BDI sustained during laparoscopic or open cholecystectomy were included. Major BDI was defined as injuries involving bile duct transection, segmental loss, ischemia, or established benign biliary stricture necessitating Roux‑en‑Y hepaticojejunostomy.

Patients with minor biliary injuries, including cystic duct stump leaks or small peripheral duct leaks managed conservatively or by endoscopic intervention, were excluded, as these do not represent the population requiring complex HPB reconstruction. Patients with traumatic or malignant biliary injuries and those lost to follow‑up prior to definitive surgery were also excluded.

Data were extracted from the prospective registry, operative records, referral documents, and electronic radiology systems. Variables recorded included patient demographics, indication for index surgery, surgeon experience at the index centre, operating environment and infrastructure, difficult gallbladder features, mechanism and timing of injury recognition, Strasberg classification, vascular involvement, referral delays, interventions attempted at the referring centre, preoperative evaluation, details of definitive repair, postoperative morbidity, and long‑term outcomes.

Descriptive statistics were used for baseline characteristics. Continuous variables are presented as mean ± standard deviation (SD), while categorical variables are presented as number (n) and percentage (%). Comparisons between categorical variables were performed using the chi-square test or Fisher’s exact test where appropriate. Continuous variables were analyzed using the independent Student’s t-test. A p-value <0.05 was considered statistically significant. Statistical analysis was performed using IBM SPSS Statistics for Windows, version 26.0 (released 2019, IBM Corp., Armonk, NY). The association between surgeon experience category and frequency of major BDI in the reconstructed cohort was assessed using the chi-square test.

## Results

A total of 23 patients with major iatrogenic BDI requiring Roux-en-Y hepaticojejunostomy were included in the reconstructed cohort. The mean age of the patients was 44.3 ± 14.2 years (range 21-68 years). Most injuries occurred during LC (21/23, 91.3%), while two injuries (8.7%) occurred during open cholecystectomy. Difficult gallbladder features were documented in 17 of 23 patients (73.9%).

The demographic characteristics, injury patterns, and perioperative variables of the reconstructed cohort are summarized in Table 1, while the relationship between surgeon experience and intraoperative management patterns is presented in Table 2. Intraoperative recognition of BDI occurred in 15 of 23 patients (65.2%), whereas the remaining 8 patients (34.8%) were diagnosed postoperatively following bile leak, sepsis, bilioma, or jaundice (Table 1).

**Table 1:** Demographic and index injury characteristics of patients with major bile duct injury. Data are presented as number/total (n/N) and percentage (%) for categorical variables, mean ± standard deviation (SD) for normally distributed continuous variables, and median (interquartile range (IQR)) for non-normally distributed continuous variables. Comparisons between categorical variables were performed using the chi-square test or Fisher’s exact test where appropriate. A p-value <0.05 was considered statistically significant. Percentages are calculated using n = 23 patients who underwent definitive Roux-en-Y hepaticojejunostomy. Laparoscopic-to-open conversion is included within laparoscopic cholecystectomy and not analyzed as a separate index procedure.

Variable		Value/number
Age (years), mean ± SD		44.3 ± 14.2
Sex	Male	10 /23 (43.5%)
	Female	13 /23 (56.5%)
Index procedure*	Laparoscopic cholecystectomy	21/23 (91.3%)
	Open cholecystectomy	2/23 (8.7%)
Difficult gallbladder documented at index surgery, yes		17/23 (73.9%)
Bile duct injury recognition	Intraoperative	15 /23 (65.2%)
	Postoperative period	8 /23 (34.8%)
Persistent biliary fistula (>4 weeks) after index surgery, yes		3 /23 (13.0%)
Time to referral, after index surgery	≤72 hours	2 /23 (8.7%)
	>72 hours	21/23 (91.3)
Strasberg injury distribution:	E1–2	10 /23 (43.5%)
	E3–5	13 /23 (56.5%)
Prolonged endoscopic management prior to referral, yes, n/N (%)		2/23 (8.7%)
Time interval to hepaticojejunostomy (weeks), median (IQR)		16 (12.25–17.75)
Operative time for definitive reconstruction (minutes), mean ± SD		181 ± 43
Intraoperative lowering of hilar plate for Hepaticojejunostomy, yes		2/23 (8.7%)
Postoperative hospital stays (days), mean ± SD		8.6 ± 2.6
Superficial surgical site infection, yes		4/23 (17.4%)
Follow-up duration after hepaticojejunostomy (months), mean ± SD		27.0 ± 13.7
Elevated ALP/GGT during follow-up, yes, n/N (%)		3/23 (13%)
Incisional hernia		2 /23 (8.7%)

**Table 2:** Surgeon experience, intraoperative management patterns, and outcomes in major bile duct injury. Categorical variables are presented as number/total (n/N) and percentage (%). Comparisons between surgeon experience groups were performed using the chi-square test or Fisher’s exact test where appropriate. A p-value <0.05 was considered statistically significant. *Three additional patients with catastrophic portal vein and hepatic artery injury died within seven days of cholecystectomy before definitive Roux-en-Y hepaticojejunostomy and were excluded from the reconstructed cohort (n = 23).

Parameter	<3 years post-training	≥3 years post-training	Overall
Number of bile duct injuries, n/N (%)	17/23 (73.9%)	6/23 (26.1%)	23/23 (100%)
Conversion to open surgery with attempted repair at index procedure, n/N (%)	10/17 (58.8%)	1/6 (16.7%)	11/23 (47.8%)
High-grade injury (Strasberg ≥E3) among attempted repairs, n/N (%)	7 / 10 (70.0%)	Not applicable	7 / 10 (70.0%)
Subtotal cholecystectomy used as bailout strategy, n/N (%)	0/17 (0%)	0/6 (0%)	0/23 (0%)
Availability of intraoperative ICG at index centres, n/N (%)	0/17 (0%)	0/6 (0%)	0/23 (0%)
Mortality prior to definitive reconstruction*, n	3	0	3
Distribution of major BDI by surgeon experience	17/23 (73.9%)	6/23 (26.1%)	p = 0.028

A significant proportion of injuries were attributable to surgeons in the early post-training period; 17 of 23 injuries (73.9%) occurred in procedures performed by surgeons with less than three years of post-training experience, compared with 6 injuries (26.1%) caused by more experienced surgeons (p = 0.028) (Table 2).

Primary repair at the index centre was attempted in 10 of 23 patients (43.5%), most commonly after conversion to open surgery. Among these patients, seven of 10 (70.0%) were subsequently classified as having high-grade injuries (Strasberg type E3 or higher) on detailed evaluation prior to reconstruction (Table 2).

Referral to the tertiary HPB centre was frequently delayed; only two of 23 patients (8.7%) were referred within 72 hours of the index surgery, while 21 patients (91.3%) were referred after 72 hours (Table 1). None of the patients underwent subtotal cholecystectomy as a bailout strategy during the index operation, and intraoperative indocyanine green (ICG) fluorescence cholangiography was not available in any of the referring centres (Table 2).

Preoperative evaluation at our centre included liver function tests, contrast-enhanced computed tomography angiography, and magnetic resonance cholangiopancreatography in all patients. Computed tomography angiography was used to assess associated vascular injury, intra-abdominal collections, and abscess formation, whereas magnetic resonance cholangiopancreatography defined the level of biliary injury and ductal anatomy before reconstruction. At referral, jaundice was present in 16/23 patients (69.56%). Postoperative diagnosis at the referring centre had occurred in 8/23 patients (34.8%), presenting with bile leak, sepsis, bilioma, or jaundice.

Definitive reconstruction was performed according to institutional policy after a delay of more than eight weeks from the index cholecystectomy. The median interval between index cholecystectomy and Roux-en-Y hepaticojejunostomy was 16 weeks (interquartile range 12.25-17.75 weeks) (Table 1). Two patients (8.7%) had prolonged endoscopic management prior to referral, undergoing 10-12 sessions of endoscopic dilatation and stenting with recurrent cholangitis. Three patients (13.0%) developed persistent biliary fistula after the index cholecystectomy and were managed with external drainage before referral. In two patients (8.7%), intraoperative lowering of the hilar plate was required to facilitate identification of the bile duct and construction of the hepaticojejunostomy (Table 1).

The mean operative time for definitive reconstruction was 181 ± 43 minutes, and the mean postoperative hospital stay was 8.6 ± 2.6 days (Table 1). No internal or external biliary stents were placed at the time of reconstruction. Postoperative morbidity was limited, with superficial surgical site infection occurring in four patients (17.4%), all managed by opening of sutures, and no deep surgical site infection or anastomotic leak. Two patients (8.7%) subsequently developed incisional hernia during follow-up (Table 1). Histopathology reports were available in 21 of 23 patients (91.3%) and confirmed benign disease in all cases. The mean duration of follow-up after hepaticojejunostomy was 27.0 ± 13.7 months. All reconstructed patients were clinically well at the last follow-up. Follow-up surveillance included liver function tests every three months and abdominal ultrasonography every six months, with magnetic resonance cholangiopancreatography performed only when clinically indicated. During follow-up, three patients (13.0%) developed elevated alkaline phosphatase and gamma-glutamyl transferase levels; however, magnetic resonance cholangiopancreatography demonstrated no evidence of anastomotic stricture in any of these cases.

In addition to the reconstructed cohort, three patients with catastrophic vasculobiliary injury involving the portal vein and hepatic artery were referred during the study period but died before definitive reconstruction and were therefore excluded from the reconstructed cohort (Table 2).

## Discussion

BDI remains one of the most serious complications of LC, with far-reaching consequences including recurrent cholangitis, secondary biliary cirrhosis, and impaired long-term quality of life [15].

Current international guidance underscores that prevention is driven primarily by disciplined operative strategy, namely, strict adherence to the Critical View of Safety, early recognition of hazardous anatomy such as false infundibulum or a concealed cystic duct, and timely adoption of bail-out techniques when safe dissection cannot be ensured [16-18]. 

The present study provides a real-world snapshot of major BDI requiring definitive HPB reconstruction in 2025 from a government tertiary referral centre in a Sub-Himalayan region. Unlike many published series that originate from high-volume apex centres, this cohort reflects injuries sustained predominantly in peripheral and resource-limited settings, thereby offering important insights into the circumstances under which major BDIs continue to occur and the factors that determine their eventual outcome.

A consistent and striking observation in this series is the strong association between early post-training surgical practice and the occurrence of major BDI.

Seventeen of 23 injuries (73.9%) were caused by surgeons with less than three years of post-training experience, compared with six injuries (26.1%) caused by more experienced surgeons. Notably, all mortality in the study occurred in this early post-training group.

The Safe Cholecystectomy Multi-Society Practice Guideline offers the most comprehensive consensus framework for prevention, while the World Society of Emergency Surgery (WSES) guideline similarly emphasizes structured preventive strategies, early diagnosis, and appropriate referral pathways as key determinants of outcome [17-20].

This finding does not imply a lack of intent or diligence but highlights a vulnerable transition phase in surgical training, where newly qualified surgeons are often required to operate independently in environments with limited supervision, high service load, and inadequate infrastructure. The consequences of this systemic gap are reflected in the severity of injuries and adverse outcomes observed.

Most major BDIs are attributable to misperception rather than technical error, with surgeons misidentifying the common bile duct or common hepatic duct as the cystic duct in a hostile Calot’s triangle, especially during acute inflammation or in the presence of contracted gallbladders, dense adhesions, and distorted anatomy. Equally important is the pattern of response following injury [11,16,18].

Primary repair at the index centre was attempted in 10 of 23 patients (43.5%), after conversion to open surgery and in association with delayed referral. This observation is clinically important, as high-grade BDIs may be accompanied by vasculobiliary involvement-most commonly right hepatic artery injury-which substantially increases operative complexity and adversely affects outcomes when unrecognized or inappropriately managed at the index center [9,14,21,22]

Among patients in whom primary repair was attempted, seven of 10 (70%) were ultimately found to have high-grade injuries (Strasberg type E3 or higher), suggesting that limited or non-definitive repair attempts in resource-constrained settings may contribute to escalation of injury severity and increased complexity of subsequent reconstruction

These findings reinforce existing evidence that non-specialist repair of major BDI, particularly in high-grade injuries, risks escalation of injury severity and compromises definitive reconstruction. The three fatal biliovascular injuries in this series underscore the catastrophic consequences of such salvage attempts, especially when vascular structures are involved [23].

A key practice-changing message from contemporary guidelines is that non-specialist repair of BDI is linked to poorer outcomes; early referral to an HPB center is therefore fundamental to optimal care rather than optional [19,24,25].

The absence of subtotal cholecystectomy as a bailout strategy in difficult gallbladder scenarios represents another critical observation. Although difficult gallbladder anatomy was documented in 17 of 23 patients (73.9%), subtotal cholecystectomy was not employed in any case.

This finding points toward a persistent gap in intraoperative decision-making rather than a technical limitation. Subtotal cholecystectomy is a well-established and safe alternative that permits completion of the procedure while avoiding hazardous dissection in the hepatocystic triangle and warrants stronger emphasis during surgical training and the early independent practice period [11,26-28].

Infrastructure limitations further compounded the risk of injury in this cohort. None of the hospitals where the index BDI occurred had access to intraoperative ICG fluorescence cholangiography.

Although ICG does not eliminate the risk of BDI, its absence in high-risk operative settings places surgeons at a significant disadvantage, particularly during difficult dissections. Expecting consistently safe outcomes without access to contemporary visualization adjuncts is unrealistic, and system-level investment in operating room infrastructure must progress in parallel with reforms in surgical training and supervision [29-31].

All available histopathology in this cohort confirmed benign disease, underscoring that the observed morbidity, mortality, and long-term sequelae were entirely iatrogenic in origin. Importantly, despite delayed referral and complex injury patterns, patients who underwent definitive reconstruction and survived demonstrated favorable medium-term outcomes, with satisfactory biliary drainage and no radiological evidence of anastomotic stricture during follow-up. These findings highlight the efficacy of delayed reconstruction when performed in specialized HPB centres.

This study also brings attention to referral bias and under-reporting. The cases analyzed likely represent only a small proportion of the true burden of major BDI in the region, as fatal injuries, locally managed complications, and cases referred to tertiary centres outside the state remain absent from most datasets.

This under-ascertainment has important implications for health policy formulation, surgical training oversight, and the development of effective quality assurance mechanisms.

This study is limited by its retrospective design and relatively small sample size, which preclude robust multivariate analysis. Nevertheless, the consistency of observed patterns and the strength of clinically meaningful associations render the findings valuable as hypothesis-generating data and directly relevant to surgical training, patient safety, and health-system design.

In summary, major BDI in 2025 remains largely preventable. The findings of this study suggest that improving outcomes will require not only technical training but also structured supervision during early independent practice, acceptance of bailout strategies such as subtotal cholecystectomy, avoidance of inappropriate primary repair attempts, timely referral to HPB centres, and upgrading of infrastructure to include modern adjuncts such as ICG fluorescence. Addressing these factors is essential to reducing preventable morbidity and mortality associated with BDI.

## Conclusions

In this five-year tertiary referral series, major iatrogenic BDI occurred predominantly during LC, usually in difficult gallbladders, and most often in procedures performed by surgeons with less than three years of post-training experience. Early non-specialist repair attempts and delayed referral were common. Three patients with catastrophic vasculobiliary injury died before definitive reconstruction. Among the patients who underwent delayed Roux-en-Y hepaticojejunostomy at our centre, postoperative and medium-term outcomes were favorable. These findings support early recognition, avoidance of unsafe repair attempts, use of bailout strategies such as subtotal cholecystectomy in difficult cases, and prompt referral to specialist HPB centres.
